# Performing a knee arthroscopy among patients with degenerative knee disease: one-third is potentially low value care

**DOI:** 10.1007/s00167-021-06615-7

**Published:** 2021-06-19

**Authors:** T. Rietbergen, P. J. Marang-van de Mheen, R. L. Diercks, R. P. A. Janssen, H. M. J. van der Linden-van der Zwaag, R. G. H. H. Nelissen, E. W. Steyerberg, L. van Bodegom-Vos, for the SMART study group, P. Pander, D. J. Hofstee, R. C. I. van Geenen, K. L. M. Koenraadt, J. P. A. H. Onderwater, Y. V. Kleinlugtenbelt, T. Gosens, T. V. S. Klos, P. C. Rijk, B. Dijkstra, A. V. C. M. Zeegers, R. A. G. Hoogeslag, M. H. A. Huis in’t Veld, A. A. Polak, N. R. Paulino Pereira, T. M. J. S. Vervest, H. C. van der Veen

**Affiliations:** 1 Department of Biomedical Data Sciences, Section Medical Decision Making, Leiden University Medical Center, Postzone J10-s, P.O. Box 9600, 2300 RC Leiden, the Netherlands; 2 Department of Orthopaedics, University Medical Center Groningen, Groningen, the Netherlands; 3 Department of Orthopaedics, Máxima Medical Center, Eindhoven, the Netherlands; 4 Department of Orthopaedics, Leiden University Medical Center, Leiden, the Netherlands

**Keywords:** Choosing Wisely, Degenerative knee disease, Knee arthroscopy, De-implementation

## Abstract

**Purpose:**

The purpose of this study was to assess in which proportion of patients with degenerative knee disease aged 50+ in whom a knee arthroscopy is performed, no valid surgical indication is reported in medical records, and to explore possible explanatory factors.

**Methods:**

A retrospective study was conducted using administrative data from January to December 2016 in 13 orthopedic centers in the Netherlands. Medical records were selected from a random sample of 538 patients aged 50+ with degenerative knee disease in whom arthroscopy was performed, and reviewed on reported indications for the performed knee arthroscopy. Valid surgical indications were predefined based on clinical national guidelines and expert opinion (e.g., truly locked knee). A knee arthroscopy without a reported valid indication was considered potentially low value care. Multivariate logistic regression analysis was performed to assess whether age, diagnosis (“Arthrosis” versus “Meniscal lesion”), and type of care trajectory (initial or follow-up) were associated with performing a potentially low value knee arthroscopy.

**Results:**

Of 26,991 patients with degenerative knee disease, 2556 (9.5%) underwent an arthroscopy in one of the participating orthopedic centers. Of 538 patients in whom an arthroscopy was performed, 65.1% had a valid indication reported in the medical record and 34.9% without a reported valid indication. From the patients without a valid indication, a joint patient–provider decision or patient request was reported as the main reason. Neither age [OR 1.013 (95% CI 0.984–1.043)], diagnosis [OR 0.998 (95% CI 0.886–1.124)] or type of care trajectory [OR 0.989 (95% CI 0.948–1.032)] were significantly associated with performing a potentially low value knee arthroscopy.

**Conclusions:**

In a random sample of knee arthroscopies performed in 13 orthopedic centers in 2016, 65% had valid indications reported in the medical records but 35% were performed without a reported valid indication and, therefore, potentially low value care. Patient and/or surgeons preference may play a large role in the decision to perform an arthroscopy without a valid indication. Therefore, interventions should be developed to increase adherence to clinical guidelines by surgeons that target invalid indications for a knee arthroscopy to improve care.

**Level of evidence:**

IV.

## Introduction

Approximately 25% of people aged 50 years and over experience knee symptoms from degenerative knee disease [16]. Degenerative knee disease is typically the result of wear and tear of the cartilage of the knee joint. Patients with degenerative knee disease may suffer from pain and stiffness of the knee [9, 19] and experience locking, clicking, or other mechanical symptoms [16].

Clinical guidelines [1, 7, 16, 20] recommend non-surgical treatments (e.g., physical therapy, pain medication (acetaminophen, NSAIDs and opioids), and dietary advice (for weight loss)) for patients aged 50 years and over with degenerative knee disease. Arthroscopy is only warranted, in case of a truly locked knee due to an intra-articular mechanical blockage (when a patient is objectively unable to fully extend his/her knee) [16], or if pain is not reduced after non-surgical treatments [1, 7, 20]. These clinical guidelines are based on evidence showing that a knee arthroscopy for these patients has no benefit compared to non-surgical treatment [4, 8, 10, 18]. Moreover, undergoing a knee arthroscopy can cause harm for patients and waste resources [4, 14], and are, therefore, considered as low value care in Dutch Choosing Wisely recommendations and similarly by medical societies in other countries [2, 12, 21, 22].

Despite the availability of guidelines and Choosing Wisely recommendations to treat degenerative knee disease primarily with non-surgical treatments, previous studies have shown that patients worldwide are not treated accordingly [3, 11, 17]. A recent study showed, for example that 70% of the patients did not receive physical therapy and 89% did not have regular pain medication (> 2 prescriptions within 6 months) prior to a knee arthroscopy [11]. On a global scale, arthroscopic knee surgery for degenerative knee disease is performed more than two million times each year [16]. However, previous research did not assess the surgical indications for patients undergoing a knee arthroscopy, and which proportion was valid or not, with the latter suggesting potentially low value care. Insight in the extent to which such low value knee arthroscopies are performed and what reasons are reported for them, is needed to develop tailored interventions to reduce them.

Therefore, the aim of this study is to assess which proportion of performed knee arthroscopies among patients aged 50 years and over is performed without a valid surgical indication being reported and thus potentially low value care, in 13 Dutch hospitals/private clinics as well as to explore factors associated with undergoing such a low value knee arthroscopy. Based on findings in other countries showing that patients frequently do not receive non-surgical treatments before a knee arthroscopy, it is hypothesized that a considerable proportion of the performed knee arthroscopies do not have a valid indication.

## Materials and methods

The Medical Ethical Committee (CME P16.190/NV/nv) of the Leiden University Medical Center waived the need for ethical approval under Dutch law for this retrospective study using administrative data and medical record review in 13 Dutch orthopedic centers (hospitals and private clinics). The data used were collected as part of the baseline measurement of the ‘SMART (Step-down MRI’s and ARThroscopies) study’, an intervention study that aimed to reduce the use of low value MRI and arthroscopies for patients with degenerative knee disease.

Administrative data were collected and all patients aged 50 years and over with knee complaints (surgical Diagnosis Treatment Codes (DTC) 1801–1899), treated in 2016 in one of the 13 Dutch orthopedic centers, were selected. Collected data included: a unique anonymized patient ID, Diagnosis Treatment Code, type of care trajectory (initial or follow-up treatment), performed arthroscopy (yes/no), age at the start of the care trajectory. An initial care trajectory starts when a patient first visits a hospital for knee complaints. A follow-up trajectory can be opened after the initial trajectory when the patient still suffers from the same complaints. The maximum duration of an initial and follow-up trajectory is 120 days.

Each orthopedic center kept a data file which linked the unique anonymized patient IDs to the actual local patient numbers, which was not accessible for the researchers. Subsequently, the researchers randomly selected a sample of 50 patients who underwent a knee arthroscopy in each orthopedic center for retrospective chart review, and each orthopedic center looked up the patient charts matching the anonymized ID. All participating orthopedic centers gave permission and if required by hospital regulations, individual patients’ permission was asked for reviewing their medical record. From the medical records, the surgical indications for knee arthroscopy (recorded on a date preceding the knee arthroscopy) were retrieved for each patient. Based on the Dutch knee arthroscopy guideline [1] and expert opinion from an orthopedic surgeon specialized in knee problems, an arthroscopy was coded as performed for a valid or invalid surgical indication (Table 1). An arthroscopy performed without a valid indication was considered as potentially low value care. The valid indications in the category “other symptoms” were all reviewed by an orthopedic surgeon specialized in knee problems (RD) and considered as being potentially performed for a valid indication (based on the information reported in the medical record).

**Table 1:** Valid and invalid surgical indications for knee arthroscopy

Valid indications	Invalid indications
Anterior Cruciate Ligament (ACL) symptoms	Pseudo locking symptoms (‘locking’, ‘clicking’ or other mechanical symptoms without an objective extension limitation of the knee), reported in a medical record of a patient with words like ‘pseudo-locking symptoms’ and ‘locking symptoms without a real blockage’
A truly locked knee (an extension limitation of the knee due to an intra-articular blockage, e.g., a meniscal tear)	Arthroscopy performed as a result of a patient-provider decision (yes/no), defined as a decision that was made in consultation with the patient or on patient’s request. Since the study is retrospective, this can only be determined if this information was written in the patient medical record
Ineffective previous non-surgical treatment, defined as having at least physical therapy	All other cases without a valid or invalid indication reported
Symptoms caused by a traumatic moment. A traumatic moment is defined as a sport injury, cycling accident or a fall	
Other symptoms (cyst, biopsy, synovitis, loose bodies, complications after arthroscopy, complications after knee replacement, complex lesion, bucket handle lesion, bone bruise, infection)	

From the random sample of 650 patients with knee complaints aged 50 + who underwent a knee arthroscopy, only the medical records of patients with degenerative knee disease (surgical DTC 1801 “Arthrosis” and 1805 “Meniscal lesion”) were included in the analyses (*n* = 542, 83.5%). The patient selection for degenerative disease was based on surgical DTC codes 1801 and 1805 which are, according to expert opinions and a survey among Dutch orthopedic surgeons, the DTC codes used for these patients in daily practice. The minimal sample size (*n* = 335) for the medical record review was calculated using a single population proportion formula; with the assumption of 0.5 without a valid indication, acceptable margin of error 0.05, a 95% confidence level, and a total population undergoing arthroscopy of 2556.

### Statistical analysis

Descriptive statistics were used to describe the characteristics of orthopedic centers, the proportion of all patients with degenerative knee disease treated in the 13 centers who underwent an arthroscopy, the proportion knee arthroscopies in the sample of medical records reviewed that were performed with and without a valid indication, and which specific indications were reported for the performed knee arthroscopies.

A multivariate logistic regression was performed to assess the extent to which age of the patient, diagnosis (arthrosis or meniscal lesion), and care trajectory (initial or follow-up) were associated with the decision to perform a knee arthroscopy without a valid indication reported. All analyses were performed using the software package SPSS (IBM SPSS, version 23). A *p* value lower than 0.05 was considered statistically significant.

## Results

### Background characteristics

As shown in Table 2, six of the 13 participating orthopedic centers were teaching hospitals (46.2%), 3 were general hospitals (23.1%), 2 were University Medical Centers (15.4%) and 2 were private clinics (15.4%). In 2016, 31,184 patients aged 50 years and older with knee complaints visited one of the participating orthopedic centers, of whom 26,991 (86.6%) had degenerative knee disease. The number of patients with knee complaints ranged from 371 to 4538 patients across centers (median 2126, IQR [1469–3451]). Both the minimum and maximum number of patients with knee complaints were from private clinics. The percentage of patients with degenerative knee disease ranged from 75.4% in a University Medical Center to 92.5% in a teaching hospital (median 86.9%, IQR [84.9–87.4%]) (Table 2).

**Table 2:** Background characteristics per hospital (*n* = 13)

Hospital number	Type of hospital	Total number of patients with knee complaints^a^	Number of patients with degenerative knee disease^b^ (%)	Number of arthroscopies for degenerative knee disease^b^ (%)	Number of medical patient records screened for degenerative knee disease^c^	Number of medical patient records for degenerative knee disease after exclusion^c^	Number of potentially low value arthroscopies for degenerative knee disease based on medical records (%)^c^	Number of potentially low value arthroscopies for degenerative knee disease^b^ per hospital^c^
1	Teaching Hospital	3451	2968 (86.0%)	201 (6.8%)	42	42	10 (23.8%)	48 (23.8%)
2	Teaching Hospital	3297	2882 (87.4%)	370 (12.8%)	50	50	25 (50.0%)	185 (50.0%)
3	General Hospital	1469	1300 (88.5%)	87 (6.7%)	47	45	18 (40.0%)	35 (40.0%)
4	Teaching Hospital	3733	3256 (87.2%)	121 (3.7%)	41	41	12 (29.3%)	35 (29.3%)
5	Teaching Hospital	3768	3283 (87.1%)	286 (8.7%)	43	42	15 (35.7%)	102 (35.7%)
6	Private clinic	371	288 (77.6%)	73 (25.3%)	49	49	20 (40.8%)	30 (40.8%)
7	University Medical Center	461	322 (69.8%)	0 (0.0%)	–	–	–	–
8	Teaching Hospital	2126	1848 (86.9%)	245 (13.3%)	50	50	19 (38.0%)	93 (38.0%)
9	Teaching Hospital	2000	1849 (92.5%)	138 (7.5%)	41	41	10 (24.4%)	34 (24.4%)
10	Private clinic	4538	4010 (88.4%)	434 (10.8%)	47	47	13 (27.7%)	120 (27.7%)
11	General Hospital	2107	1780 (84.5%)	206 (11.6%)	50	50	14 (28.0%)	58 (28.0%)
12	General Hospital	2560	2222 (86.8%)	334 (15.0%)	44	44	28 (63.6%)	213 (63.6%%)
13	University Medical Center	1303	983 (75.4%)	61 (6.2%)	38	37	4 (10.8%)	7 (10.8%)
Total^d^	–	31184	26,991 (86.6%)	2556 (9.5%)	542	538	188 (34.9%)	893 (34.9%)

*All possible knee Diagnosis Treatment Codes (DTC) from 1801 till 1899

**Diagnosis Treatment Codes (DTC) 1801 and 1805

***Diagnosis Treatment Codes (DTC) 1801 and 1805. There was an acceptable margin of error 0.05, with a 95% confidence level

****Total of all 13 hospitals/private clinics

### Patients undergoing a knee arthroscopy

Overall, 2556 of the 26,991 (9.5%, range 0.0–25.3%) patients with degenerative knee disease underwent an arthroscopy in one of the participating orthopedic centers. One center did not perform any arthroscopy at all for patients with degenerative knee disease in 2016. From these 2556 patients, the medical records of 542 patients were reviewed on indications reported for the arthroscopy. Four patients were excluded, because the medical records reported they underwent a (total) knee replacement rather than an arthroscopy. In 350 (65.1%) of the remaining 538 patients there was at least one valid surgical indication to perform the arthroscopy reported (in total 416 indications were reported). Ineffective previous non-surgical treatment (37.3%) and a truly locked knee (33.9%) were most frequently mentioned in the medical records as an indication to perform the arthroscopy (Table 3).

**Table 3:** Valid and invalid indications to perform knee arthroscopy in degenerative knee disease

Valid indications knee arthroscopy	Amount, *n* (%)	Invalid indications (low value) knee arthroscopy	Amount, *n* (%)
ACL symptoms	13 (3.1%)	Pseudo locking symptoms	9 (4.7%)
Locking symptoms	141 (33.9%)	Arthroscopy performed as a result of a patient–provider decision	50 (26.3%)
Failed previous non-surgical treatment	155 (37.3%)	All other cases without a valid or invalid indication reported	131 (68.9%)
Symptoms caused by traumatic moment	46 (11.1%)		
Other (cyst, biopsy, synovitis, loose bodies, complications after arthroscopy, complications after knee replacement, complex lesion, bucket handle lesion, bone bruise, infection)	61 (14.7%)		
Total	416	Total	190

In 188 (34.9%) of the 538 patients there was no valid indication reported to perform a knee arthroscopy. The percentage of patients without a reported valid indication and, therefore, potentially low value care, ranged between 10.8% (in a University Medical Center) and 63.6% (in a general hospital) (Table 2). In 4.7% of these patients without a valid indication, pseudo locking symptoms were reported and patient–provider decisions (including on patient request) in 26.3% of patients (Table 3). Performing an arthroscopy without a valid indication was not associated with age [OR 1.013 (95% CI 0.984–1.043)], diagnosis [OR 0.998 (95% CI 0.886–1.124)] nor type of care trajectory [OR 0.989 (95% CI 0.948–1.032)].

## Discussion

This study shows that while for a considerable part (35%) of the knee arthroscopies performed in patients with degenerative knee disease no valid surgical indication was reported and could thus be considered potentially low value care, confirming our initial hypothesis, this also means that the majority (65%) was performed with a valid indication reported. Potentially low value knee arthroscopies were performed in all types of hospitals. A frequently reported reason (for 26% of the patients) was that it was a joint patient–provider decision or that the arthroscopy was performed on the patient’s request. Age, diagnosis and type of care trajectory were not associated with patients undergoing a potentially low value knee arthroscopy.

Previous studies have shown that a considerable number of patients with degenerative knee disease received an arthroscopy without first performing non-surgical treatments as recommended by clinical practice guidelines [3, 11, 17]. Muheim et al. [11] for instance showed that 70% of the patients in their study did not receive physical therapy before they underwent a knee arthroscopy. However, it was unknown whether the indication to perform a knee arthroscopy in these patients was valid (e.g., a truly locked knee). The results of the current study thus add to the literature that in 65% of the patients with degenerative knee disease who undergo arthroscopy there is a valid indication. In addition, from the 35% of patients without a valid indication for their knee arthroscopy, our study showed that for 26% of these patients this was the result of a patient–provider decision. Although the Choosing Wisely campaign encourages physicians and patients to engage in conversations about unnecessary tests, treatments and procedures [5], it can be questioned whether low value treatments should be considered by orthopedic surgeons as result of a patient–provider decision. If we believe the harms and costs of arthroscopies outweigh any benefits and we are trying to reduce low value care, orthopedic surgeons should practice evidence-based decision making in which they clearly explain the evidence, listen to a patient’s values and preferences, and not offer to perform an arthroscopy unless there is a valid surgical indication [15]. As already shown in a previous study of Rietbergen et al. [13], such evidence-based decision making may be hampered by orthopedic surgeons’ beliefs in the added value of arthroscopies as well as positive experiences with knee arthroscopies among friends and family in the patient’s environment.

Based on the results of the current study, 35% of the knee arthroscopies in these 13 Dutch hospitals potentially do not add value for patients with degenerative knee disease, could possibly cause harm and waste resources. Data of Dutch Hospital Data show that in 2016 12,374 knee arthroscopies (based on 61 hospitals but without any private clinics) were performed for degenerative knee disease [6]. Assuming that one third of these knee arthroscopies are potentially low value, this means that more than 1.6 million euros were spent on these knee arthroscopies in 2016 [23]. Therefore, it remains important to advocate increased adherence to clinical guidelines by surgeons and to develop interventions that target invalid indications for a knee arthroscopy.

This study also has limitations. First, the hospitals participating in this study were likely a non-representative sample of Dutch hospitals and private clinics. They have participated voluntarily in the SMART study and agreed to the retrospective review of medical records, which may have resulted in selection bias, e.g., because of interest in this issue, and could mean that these are conservative estimates if other hospitals would for instance have strong beliefs in the added value of arthroscopy. The second limitation is that the results are based on information written in medical records. It can only be assumed that all important information about indications for a knee arthroscopy were in fact reported. If documentation was incomplete, the frequency of potentially low value knee arthroscopies may have been overestimated if these were performed for valid surgical indications but not recorded. However, incomplete documentation could also have underestimated pseudo-locking symptoms and arthroscopies performed due to patient–provider decisions and thereby potentially low value knee arthroscopy, if these were not routinely reported which seems likely given that these are not valid indications for performing a knee arthroscopy according to clinical guidelines and corresponding literature. A third limitation is that we only examined the indication for which the knee arthroscopy was conducted based on the clinical guideline and corresponding literature. However, including clinical outcomes for patients after knee arthroscopies or whether patients have perceived the surgery to have had added value (e.g., reassured them) could have changed the results, both whether those considered as low value indeed did not add value for them but also whether the potentially high-value knee arthroscopy also resulted in, e.g., better patient outcomes. However, that would be a different study rather than a limitation of the way this study is conducted, and since it is not included in the guidelines what would be considered sufficient improvement or not, including patient’s outcomes and their perspective may also introduce subjectivity and inter-rater variability in the assessment as well as hindsight bias.

## Conclusions

In a random sample of patients with degenerative knee disease aged 50 years and over who underwent an arthroscopy, 65% had valid indications reported in the medical records but 35% were performed without a valid indication reported and, therefore, potentially low value care, inconsistent with clinical guidelines. Patient and/or surgeons preference may play a large role in the decision to perform an arthroscopy without a valid indication. Therefore, interventions should be developed to increase adherence to clinical guidelines by surgeons that target invalid indications for a knee arthroscopy to improve care. Objective patient information should be provided to support and improve the patient–provider decision making process.

## Abbreviations

MRI: Magnetic resonance imaging
CW: Choosing Wisely
DTC: Diagnosis Treatment Code
ACL: Anterior Cruciate Ligament

## References

[1] Arthroscopy of the knee (in dutch) (2019) https://richtlijnendatabase.nl/richtlijn/artroscopie_van_de_knie/startpagina_-_artroscopie_van_de_knie.html.

[2] Australian Rheumatology Association: tests, treatments and procedures clinicians and consumers should question (2018) http://www.choosingwisely.org.au/recommendations/ara

[3] Bergkvist D, Dahlberg LE, Neuman P, Englund M (2016). Knee arthroscopies: who gets them, what does the radiologist report, and what does the surgeon find? An evaluation from southern Sweden. Acta Orthop.

[4] Brignardello-Petersen R, Guyatt GH, Buchbinder R, Poolman RW, Schandelmaier S, Chang Y (2017). Knee arthroscopy versus conservative management in patients with degenerative knee disease: a systematic review. BMJ Open.

[5] Choosing Wisely. https://www.choosingwisely.org/

[6] Dutch Hospital Data. https://www.dhd.nl/Paginas/home.aspx

[7] ESSKA Meniscus Consensus Project: Degenerative meniscus lesions (2016) https://cdn.ymaws.com/www.esska.org/resource/resmgr/Docs/2016-meniscus-consensus-proj.pdf

[8] Khan M, Evaniew N, Bedi A, Ayeni OR, Bhandari M (2014). Arthroscopic surgery for degenerative tears of the meniscus: a systematic review and meta-analysis. CMAJ.

[9] Lazic S, Boughton O, Hing C, Bernard J (2014). Arthroscopic washout of the knee: a procedure in decline. Knee.

[10] Moseley JB, O'Malley K, Petersen NJ, Menke TJ, Brody BA, Kuykendall DH (2002). A controlled trial of arthroscopic surgery for osteoarthritis of the knee. N Engl J Med.

[11] Muheim LLS, Senn O, Fruh M, Reich O, Rosemann T, Neuner-Jehle SM (2017). Inappropriate use of arthroscopic meniscal surgery in degenerative knee disease. Acta Orthop.

[12] Orthopaedics: Ten Things Physicians and Patients Should Question (2018) https://choosingwiselycanada.org/orthopaedics/

[13] Rietbergen T, Diercks RL, Anker-van der Wel I, van den Akker-van Marle ME, Lopuhaa N, Janssen RPA (2019). Preferences and beliefs of Dutch orthopaedic surgeons and patients reduce the implementation of "Choosing Wisely" recommendations in degenerative knee disease. Knee Surg Sports Traumatol Arthrosc.

[14] Scott IA, Duckett SJ (2015). In search of professional consensus in defining and reducing low-value care. Med J Aust.

[15] Shared decision making (2019) https://rnzcgp.org.nz/GPPulse/Sector_news/2019/Shared_decision_making.aspx?WebsiteKey=5d6ca365-aa40-4574-8861-3f40caea2dce

[16] Siemieniuk RAC, Harris IA, Agoritsas T, Poolman RW, Brignardello-Petersen R, Van de Velde S (2017). Arthroscopic surgery for degenerative knee arthritis and meniscal tears: a clinical practice guideline. BMJ.

[17] Smith L, Barratt A, Buchbinder R, Harris IA, Doust J, Bell K (2020). Trends in knee magnetic resonance imaging, arthroscopies and joint replacements in older Australians: still too much low-value care?. ANZ J Surg.

[18] Thorlund JB, Juhl CB, Roos EM, Lohmander LS (2015). Arthroscopic surgery for degenerative knee: systematic review and meta-analysis of benefits and harms. Br J Sports Med.

[19] Turkiewicz A, Gerhardsson de Verdier M, Engstrom G, Nilsson PM, Mellstrom C, Lohmander LS (2015). Prevalence of knee pain and knee OA in southern Sweden and the proportion that seeks medical care. Rheumatology (Oxford).

[20] VA/DoD Clinical Practice Guideline For The Non-Surgical Management of Hip and Knee Osteoarthritis (2014) https://www.healthquality.va.gov/guidelines/CD/OA/VADoDOACPGFINAL090214.pdf

[21] Verstandige keuzes binnen de orthopedie. https://www.demedischspecialist.nl/sites/default/files/Verstandige%20Keuzes%20NOV_definitief.pdf

[22] Wisely C (2019) American Medical Society for Sports Medicine: Five Things Physicians and Patients Should Question. http://www.choosingwisely.org/societies/american-medical-society-for-sports-medicine/

[23] Zorginstituut Nederland (2014) Zinnige Zorg Verbetersignalement Artrose knie/heup

